# The Effects of Gluten on Weight Gain, Hematological, Biochemical, and Various Endocrinological Parameters

**DOI:** 10.5152/tjg.2024.23210

**Published:** 2024-03-01

**Authors:** Atilla Bektaş, Meltem Ulusoy, Levent Özsarı, Ahmet Melih Özel

**Affiliations:** 1 Department of Gastroenterology, Private Ankara Surgery Medical Center, Ankara, Turkey; 2 Division of Applied Biology, Department of Biology, Hacettepe University, Ankara, Turkey; 3 Department of Endocrinology and Metabolic Diseases, Sultan 2. Abdulhamid Khan Educational and Research Hospital, İstanbul, Turkey; 4 Department of Gastroenterology, Anadolu Medical Center Hospital, İstanbul, Turkey

**Keywords:** Gluten, gluten-free diet, weight gain, diabetogenic, glycemia, thyroid functions

## Abstract

**Background/Aims:**

This study is aimed to compare the effects of nutrition which has been enriched with different amounts of gluten to gluten-free diets on weight gain, diabetogenic state, hematological, and biochemical parameters.

**Materials and Methods:**

A total of 40 newly weaned male Wistar albino rats used in the study were randomized into 4 different groups based on the gluten rations they were given. Following 12 weeks of diet they were killed and intracardiac blood samples were collected. Groups were identified as group 1 (n = 10): control group; normal rat ration containing wheat, group 2 (n = 10): gluten-free diet, group 3 (n = 10): ration containing medium level of gluten (normal rat diet + 6% vital gluten) and group 4 (n = 10): ration containing high level of gluten (normal rat diet + 12% vital gluten).

**Results:**

In groups 3 and 4, high-density lipoprotein was found to be higher than the other 2groups. However, when group 2 results were compared to the other groups; the highest T3, T4, creatinine and B12 levels and the lowest gluten-specific IgE level were observed. alanine aminotransferase and aspartate aminotransferase levels were found to be higher in group 1 compared to the other 3 groups. No statistically significant difference was detected between the groups in terms of other parameters.

**Conclusion:**

This study provides evidence that a gluten-containing diet does not cause weight gain, has no diabetogenic effect, and also does not adversely affect general health in relation to hematological, biochemical, and various endocrinological parameters.

**Main Points**

- There was no difference between the gluten-free group and the other groups in terms of weight gain, serum glucose, and insulin levels.
- Diets containing moderate and high levels of gluten had a positive effect by raising high-density lipoprotein levels.
- It was found that T3, T4, creatinine, and vitamin B12 levels of the gluten-free diet group were higher than the other groups.

## Introduction

Gluten is a vegetable protein found in cereals, such as wheat, barley, and rye, and it consists of glutenin and gliadin fractions. Gluten is primarily associated with celiac disease (CD), nonceliac gluten sensitivity, and wheat allergy, accounting for an incidence of 1%, 0.63%-6%, and 0.1%-0.6%, respectively.^1-3^

Within the last 30 years, the gluten-free diet (GFD) market has grown disproportionately in relation to the incidences of gluten-associated diseases. Currently, several food companies create a health halo effect, such as GFD, with their preliminary information on the package information on their product packaging, leading to manipulation of consumer preferences.^4^ In 2016, more than $15.5 billion was spent for retail sales of gluten-free foods, which was double the amount spent in 2011. A 2013 study found that 65% of American adults believe that gluten-free foods are healthier, and 27% choose gluten-free products to aid in weight loss.^5,6^ However, cereals are affordable and easily accessible, and they are one of the main components of the Mediterranean diet, which is considered one of the healthiest diets worldwide.^7^ In the Mediterranean, the mean daily gluten consumption is particularly high (approximately 20 g, and even higher in some other countries).^2,7,8^

What is the validity of the claims that a gluten-containing diet could lead to certain health problems? CD and type 1 diabetes (T1D) are closely correlated. In a small-scale study, patients with T1D and CD were shown to benefit from a GFD in avoiding certain diabetes complications.^5^ However, whether a GFD is beneficial for patients without CD is unknown.^5,9^

The link between type 2 diabetes (T2D), metabolic syndrome, and the development of CD has not been elucidated.^5^ CD is also associated with other autoimmune diseases (AIDs), particularly endocrine disorders, such as T1D and autoimmune thyroiditis.^10^

However, in more than 100 AIDs, strict gluten avoidance is recommended only in gluten-associated conditions, such as CD, dermatitis herpetiformis, and gluten ataxia. Nevertheless, a GFD is not recommended in other AIDs due to a lack of well-controlled, double-blind, cross-sectional, and long-term studies.^9^

CD occurs only in genetically susceptible individuals. HLA-DQ2 (~95%) or HLA-DQ8 (~5%) are present in most patients with CD.^11^ The only effective treatment for CD is GFD, which is reported in 1% of the population.^3^

Currently, no evidence supports that a GFD has a beneficial effect on weight gain, diabetogenic effects, hematological, biochemical, and various endocrinological parameters in individuals without CD. Additionally, the effect of gluten on liver and thyroid functions in those without CD is also not fully known. Therefore, this study aimed to investigate weight gain, diabetogenic status, and changes in hematological, biochemical, and various endocrinological parameters in rats fed with a GFD and diets enriched with varying amounts of vital gluten. Thus, 40 newly weaned male Wistar albino rats were randomly divided into 4 groups of 10, and each group was fed 4 different diets based on their gluten content (standard diet, GFD, 6% vital gluten-supplemented diet, and 12% vital gluten-supplemented diet). After 12 weeks, the rats were killed, and intracardiac blood samples were collected and analyzed.

## Materials and Methods

This study was approved by Başkent University Ethical Committee for Experimental Research on Animals (Project no: DA18/31) and was conducted at the Laboratory Animal Production and Research Center of Başkent University. All animal experiments followed the principles of animal care (NIH publication no. 85–23, revised 1985) and the national laws on the Protection of Animals.^12^

### Rats

Forty male Wistar albino that were weaned at 3 weeks were used. After the completion of breastfeeding, the rats were divided into 4 groups by using a simple random sampling method and were fed 1 of 4 different amounts of gluten-containing rations for 12 weeks. During this period, the rats had free access to food and acidified drinking water. They were kept at a room temperature of 20-22°C with a 12-hour light–12-hour darkness (12L : 12D) cycle. 

### Experimental Design

In this study, the rats were randomly divided into 4 different groups.^13^

Group 1 (control group, n = 10) was fed with standard rat ration containing 22% total protein and 2901 kcal/kg.

Group 2 (GFD group, n = 10) was fed with a gluten-free ration containing 22% total protein and 3165 kcal/kg. The diet did not contain wheat protein and corn starch.

Group 3 (normal/standard rat diet + 6% vital supplemented gluten, n = 10): rats were fed with a ration containing medium gluten, 22% total protein, and 3075 kcal/kg.

Group 4 (normal/standard rat diet + 12% vital supplemented gluten, n = 10) was fed with a ration containing high gluten, 22.8% total protein, and 3157 kcal/kg. The diet did not include corn starch (Table 1).

Their weekly weight gain and daily–weekly feed consumption data were collected. After 12 weeks of diet, the rats were killed by decapitation under intraperitoneal xylazine and ketamine anesthesia, and intracardiac blood samples were collected.

### Ration Preparation

In the gluten-free group, the gluten-containing ingredients were replaced with meat protein while maintaining a similar content of protein, fat, fiber, minerals, and energy. Otherwise, in the medium and high gluten groups, meat protein was replaced with vital supplemented gluten while keeping the same content of wheat, soybean, milk, and meat protein. All group diets were equally supplemented with vitamins, minerals, and amino acids (Table 1). In this study, the protein (gluten) content of wheat was evaluated as 80%.^12^

We set the isocaloric nutrition provided to the rats at 2900 kcal with breeding and maintenance feeds suitable for the growth period. The feeds were given ad libitum and designed as isonitrogenic to prevent the development of differences between the rats. The rats received natural chow, classified as maintenance and breeding foods, which provided 22.0%-22.8% protein, 2901-3165 kcal, and 5% cellulose. Although the rats in group 1 were fed with natural feed, specially prepared feeds were used for the other groups. When the feed preparation for the gluten-free group (group 2) was completed, the feeds for groups 1, 3, and 4 were prepared similarly but with different gluten amounts (Table 1).

### Hematological and Serum Biochemical Analyses

The blood samples were separated using a serum separator tube and allowed to coagulate at room temperature for 2 hours or overnight at 2-8°C. The samples were centrifuged at 1000 ×*g* (or 3000 rpm) for 15 minutes. The sera were immediately aliquoted, and were stored at −20°C or −80°C.

C-reactive protein (CRP), fasting blood glucose, fasting insulin, liver function tests, lipid profile, urea, creatinine, thyroid autoantibodies, thyroid function, vitamin B12, ferritin, folic acid, and gluten-specific Ig E levels were analyzed in this study. A complete blood count was used for hemogram (Sysmex XN2000, Sysmex, Germany).

Serum glucose, urea, creatinine, alanine aminotransferase (ALT), aspartate aminotransferase (AST), alkaline phosphatase (ALP), total cholesterol (TC), triglyceride, low-density lipoprotein (LDL), high-density lipoprotein (HDL), very-low-density lipoprotein (VLDL), and CRP concentrations were determined using Beckman AU5811 (Beckman, California, USA ) commercial kits. Specific gluten IgE concentration was measured using F79 Gluten Siemens Immulite 2000 (Siemens, Germany) commercial kit. Ferritin, folic acid (B9), vitamin B12, free triiodothyronine (FT3), free thyroxine (FT4), thyroid-stimulating hormone (TSH), anti-thyroglobulin (anti-Tg), and anti-thyroid peroxidase (anti-TPO) levels were determined using Abbott İ2000 (Abbott, USA ) commercial kits. The insulin level was analyzed using an enzyme-linked immunosorbent assay.

### Statistical Analysis

Statistical analysis was performed using the Number Cruncher Statistical System software (NCSS 2007, Kaysville, Utah, USA). Study data were compared using the Kruskal–Wallis test for quantitative variables (mean, standard deviation, median, etc.), and the Bonferroni–Dunn test was used for subgroup comparisons (post hoc assessment).

## Results

In this study, the rats were fed gluten-free and gluten-containing diets throughout their lives. The rats in all groups remained healthy throughout the experiment. 

**Figure 1.**
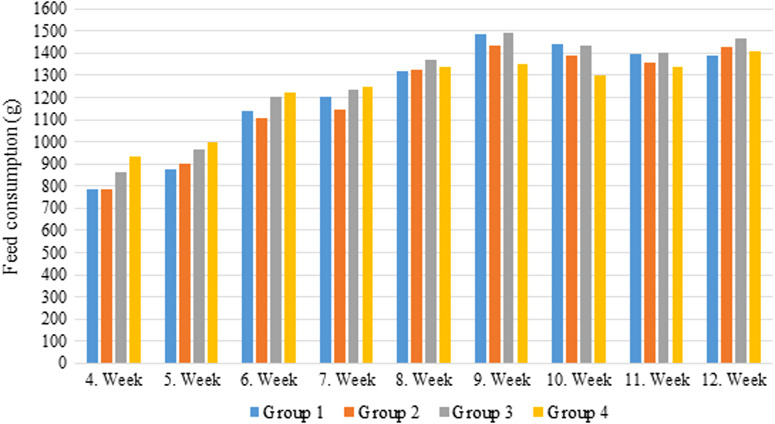
Mean weekly feed consumption of the rats in each group.

**Figure 2.**
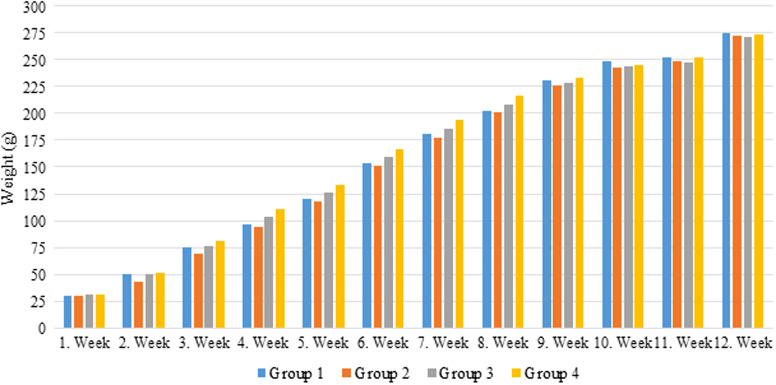
Weekly mean cumulative body weight gain.

Although feed consumption and weight gain were high between the third and eighth weeks in groups 3 and 4, the weight gain difference between the groups was not statistically significant (Figures 1 and 2).

The differences in insulin, serum glucose, TC, LDL, VLDL, triglyceride, urea, folic acid, ferritin, TSH, anti-Tg, anti-TPO, and CRP levels were not statistically significant between the groups. Moreover, hematological parameters were not statistically different between the groups.

The difference between the mean T3 values of the rats in groups 2 and 3 (*P*_2-3_: .001; *P *< .01) was statistically significant. According to the pairwise comparison results, there was a statistically significant difference in T4 levels between groups 2 and 1 and between groups 2 and 3 (*P*_2-1_: .006; *P*_2-3_: .006; *P *< .01) (Table 2). In addition, the T3 and T4 levels in the GFD group were higher than those in the other groups.

The mean serum gluten-specific Ig E level in the rats in the GFD group was lower than that of the other groups (*P*_2-1_: .005, *P*_2-3_: .005, *P*_2-4_: .005; *P *< .01) (Table 2), and their difference was statistically significant.

The mean serum vitamin B12 and creatinine levels of the rats in the GFD group were higher than those of the other groups. In the pairwise comparison statistical analyses, the difference between the vitamin B12 level of the GFD group and other groups was significant (*P*_2-1_: .001, *P*_2-3_: .001, *P*_2-4_: .001; * P *< .01). The difference between the creatinine levels of the GFD group and group 4 was also significant (*P*_2-4_: .011; *P *< .001) (Table 2).

The mean serum HDL levels of the rats in groups 3 and 4 were higher than those of the rats in other groups. According to the pairwise comparison results, a statistically significant difference was found between groups 3 and 1 and between groups 3 and 2 in HDL levels (*P*_3-1_: .003, *P*_3-2_: .003; *P* < .01) (Table 2).

The serum ALT and AST levels of the rats in group 1 were higher than those of the rats fed with a gluten-free ration and rations containing different amounts of vital gluten (*P*_1-3_: .001, *P*_1-4_: .001; *P *< .01). The difference between the mean AST levels of the rats in groups 1 and 4 was statistically significant (*P*_1-4_: .046; *P *< .05) (Table 2).

## Discussion

Animal model studies with vital gluten-added rations in the literature are unable to adequately represent the high amount of cereal consumption, especially because many beneficial food ingredients are present in whole grain, such as fiber, vitamins, minerals, and antioxidants, in addition to gluten.^14^ Fibers and antioxidants in cereals prevent the development of many chronic illnesses, such as T2D, cardiovascular disease, and obesity.^15^ This study showed that the ratio in group 1 contains a similar amount of wheat gluten as the mean amount in the human diet.

Some celebrities claimed GFD to cause weight loss and improve athletic performance.^4,5^ When the average weights of the rats in groups 1, 2, 3, and 4 were examined at the end of the 12th week; 274.3091 g, 272.1909 g, 20.9727 g, and 273.8273 g, respectively. Based on these findings, it can be inferred that there is no correlation between gluten consumption and weight gain (Figures 1 and 2).

The main characteristic of T1D is the autoimmune destruction of the pancreatic beta cells, leading to a lack of insulin production. In animal models, this deficiency in insulin production is achieved by different mechanisms, ranging from chemical ablation of the beta cells to breeding rodents that spontaneously develop autoimmune diabetes.^16^ However, we did not manipulate our rats in this study.

Gluten consumption is a disease-initiating factor in CD and might also have a role in T1D development. Studies performed in T1D animal models have reported that diet affects the pathogenesis. Although some studies suggest that GFD may preserve beta cell function, other studies have not found this effect.^17^

Interestingly, Funda et al^12^ showed that both gluten-free and gluten-enriched (gluten+) nonpurified diets substantially prevent diabetes in nonobese diabetic mice.

Shetty et al^18^ observed no diabetogenic effect after adding 100, 200, and 400 mg/kg pure gluten to the ration in Wistar albino rats. Therefore, removing gluten from the diet is not necessary for T2D prevention and management.

In this study, it was shown that the serum glucose/insulin ratio is 0.27, 0.27, 0.24, and 0.26 in groups 1, 2, 3, and 4, respectively, with gluten-free and normal ration groups having the highest ratio. Insulin and serum glucose levels were normal in groups 3 and 4 (Table 2). Thus, more studies with a larger population are needed to evaluate the effect of gluten on insulin resistance.

No studies in the literature focused on GFDs in nonceliac patients with Hashimoto’s disease (HD). No evidence supports that GFD is beneficial for HD. GFD does not affect the concentration of thyroid hormones, and an anti-inflammatory diet (mostly plant-based) should be implemented due to the ongoing inflammatory process in the body. CD is observed in 1% of the general population, whereas HD is 5%. Gluten should only be eliminated in patients with CD or gluten sensitivity, which may coexist with HD. GFD reduces the anti-TPO levels in patients with HD with coexisting CD. However, GFD has no effect on the TSH levels in patients with HD.^19,20^

Passali et al^21^ concluded that no evidence supports the recommendation of GFD in patients with multiple sclerosis, psoriasis, autoimmune thyroid diseases (ATDs), or T1D without CD.

In the present study, we investigated the effects of diets containing different gluten amounts on thyroid function in the absence of CD and ATD. The T3 and T4 levels in the GFD group (group 2) were higher than those in the other groups (Table 2). The TSH levels were similar in all groups. Carrier proteins in circulation store thyroid hormones. GFD may affect the binding of free T3 and T4 to thyroid-binding globulin. Thus, more studies are needed to understand the effects of GFD on thyroid hormone-binding globulin.

Liver abnormality is one of the associated extraintestinal manifestations of CD. Transaminase elevation is seen in 9% of patients with CD without any other reason. Initiating GFD in these patients may improve and normalize liver enzyme abnormality.^22^ Moghaddam et al^23^ showed that some patients with CD had reversible liver enzyme abnormality that improved with GFD. Purkins et al^24^ showed that serum transaminase activity and triglyceride concentrations substantially increased after consuming a high-calorie diet rich in carbohydrates. Katayama et al^25^ reported that high gluten consumption in male Wistar albino rats that received high-dose d-galactosamine (GalN) and endotoxin (Etx) protected against liver injury.

In the present study, ALT and AST levels in group 1 were higher than those in the gluten-free group and the groups fed with diets containing different amounts of vital gluten (Table 2). Both low TC and HDL levels are known characteristics of active CD. Treating CD using GFD may increase TC and HDL levels.^26^ GFD is associated with a high HDL level, which may have been due to the higher amount of saturated fat in GFD.^26^

In their cross-sectional study, Gnanapandithan^27^ found that GFD in subjects with and without CD did not cause a change in LDL, triglyceride, and insulin levels. Additionally, GFD is associated with high HDL levels in both CD and non-CD groups. However, they concluded that no scientific evidence showed that GFD is particularly healthy for the general population.

Elkan et al^28^ showed that the TC, LDL, and LDL/HDL ratios were significantly reduced with the administration of a vegan GFD in patients with rheumatoid arthritis; however, TG and HDL levels did not change. Another study investigated the effects of wheat fiber and gluten on the lipid profile and concluded that gluten positively contributed to the serum lipid profile independent of dietary fiber, which was consistent with other studies.^29^

In the present study, the HDL levels of the rats in groups 3 and 4 were found to be high (Table 2). Compared with the other groups, group 2 had the lowest Ig E value (Table 2), which may have been due to the absence of wheat in the diet of rats in this group. Moreover, the carbohydrate consumption in group 2 was lower than that in the other groups (Table 1). Additionally, *Aspergillus* spp., *Fusarium* spp., *Alternaria* spp., *Claviceps* spp., and similar fungi may have been consumed by the food group whose metabolite was contaminated with mycotoxins. However, performing mycotoxin analyses of feed groups in the study was not possible. 

Compared with the other groups, the GFD group showed the highest serum creatinine and vitamin B12 levels (Table 2). In this study, it was shown that the total amount of protein in the diet of all groups is 22 g. The higher serum creatinine and vitamin B12 levels in the GFD group might be because of the higher use of animal protein in this group compared with the other groups.

The difference in hematology, feed consumption, weight, folic acid, ferritin, CRP, ESR, FBG, insulin, TSH, anti-Tg, anti-TPO, TC, triglyceride, VLDL, and LDL levels was not statistically significant between the rats fed with GFD and diets containing different amounts of gluten (Table 2).

In this study, evidence that a gluten-containing diet does not cause weight gain, has no diabetogenic effect, and does not adversely affect general health in relation to hematological, biochemical, and various endocrinological parameters.

Cereals have been the basis of our diet for thousands of years and one of the main components of the Mediterranean diet, which is considered one of the healthiest diets worldwide. However, recommending GFD to the public without medical indications is not scientifically based, except in conditions such as CD. 

## Figures and Tables

**Table 1.** Composition of the Test Rations (g/100g) Modified from Funda et al^12^ Ration Arrangement Based on the Human Nutrition Model

Ration Content (g/100g)	Group 1 Normal Rat Ration	Group 2 Gluten-Free Ration	Group 3 Normal Rat Ration+6% Vital Gluten (Medium Gluten)	Group 4 Normal Rat Ration+12% Vital Gluten (High Gluten)
Total protein	22	22	22	22.8
Wheat protein* (g/100 g)	3.5	–	3.5	3.5
Soy protein (g/100 g)	6.5	6.5	6.5	5.0
Corn starch (g/100 g)	3.0	–	3.5	–
Gluten (vital supplement)	–	–	6	12.0
Meat protein (g/100 g)	8.0	14.5	1.5	1.5
Milk protein (g/100 g)	1.0	1.0	1.0	0.8
Dry matter (g/100 g)	87.76	87.76	87.76	87.76
Raw oil (g/100 g)	5.50	5.50	5.50	5.50
Fiber (g/100 g)	5.00	5.00	5.00	5.00
Mineral (g/100 g)	7.74	7.74	7.74	7.74
Lysine (g/100 g)	1.31	1.31	1.31	1.31
Methionine (g/100 g)	0.50	0.50	0.50	0.50
Threonine (g/100 g)	0.83	0.83	0.83	0.83
Tryptophan (g/100 g)	0.20	0.20	0.20	0.20
Methionine + cysteine (g/100 g)	0.86	0.86	0.86	0.86
Water	5.7	4.2	4.9	5.2
Energy (kcal/kg)	2.901	3.165	3.075	3.157

*Eighty percent of wheat protein is gluten.

**Table 2.** Mean Levels of Blood Parameters, Standard Deviations, and *P*-Values of the Rats Fed with Rations Containing Different Amounts of Gluten

	Group 1, n = 10 (Minimum–Maximum)	Group 2, n = 10 (Minimum–Maximum)	Group 3, n = 10 (Minimum–Maximum)	Group 4, n = 10 (Minimum–Maximum)	*P*
Serum glucose (mg/dL)	170.10 ± 23.89 (123-203)	169.10 ± 31.64 (112-214)	163.90 ± 19.58 (137-203)	178.10 ± 44.93 (123-238)	.913
Insulin (µU/mL)	635.355 ± 130.60 (508,5-877)	617.37 ± 140.31 (425.5-904.2)	662.13 ± 147.39 (437.8-896)	673.4 ± 69.3649 (566-792)	.750
Total cholesterol (mg/dL)	55.40 ± 6.433 (45-67)	54.50 ± 7.531 (46-65)	61.00 ± 7.972 (46-76)	60.20 ± 7.300 (47-72)	.170
LDL (mg/dL)	17.50 ± 2.068 (14-21)	17.40 ± 3.438 (13-22)	18.50 ± 3.171 (15-26)	19.20 ± 4.803 (13-30)	.818
HDL (mg/dL)	34.710 ± 4.8905 (25.7-43.9)	36.070 ± 4.6046 (31.0-41.1)	42.340 ± 5.5502 (31.2-52.3)	40.410 ± 2.9991 (34.3-43.8)	.003** Group 3 > group 1 Group 3 > group 2
VLDL (mg/dL)	16.70 ± 3.401 (8-20)	17.50 ± 3.274 (14-24)	18.90 ± 5.646 (11-29)	13.90 ± 4.909 (7-22)	.155
Triglyceride (mg/dL)	83.110 ± 17.6406 (38.5-101.7)	87.250 ± 16.9849 (68.6-118.6)	94.170 ± 28.2710 (53.9-145.3)	69.350 ± 24.5542 (33.1-109.5)	.157
Urea (mg/dL)	37.580 ± 2.9059 (30.3-40.6)	38.210 ± 4.2777 (34.1-46.2)	39.700 ± 5.5150 (32.8-52.8)	35.410 ± 6.9652 (25.3-45.7)	.385
Creatinine (mg/dL)	0.2840 ± 0.04858 (0.23-0.39)	0.3300 ± 0.03055 (0.29-0.38)	0.2830 ± 0.03466 (0.22-0.35)	0.2800 ± 0.03771 (0.23-0.37)	.011* Group 2 > group 4
ALT (U/L)	40.80 ± 6.57 (34-57)	36.10 ± 5.60 (29-49)	32.60 ± 3.62 (27-39)	31.90 ± 3.90 (28-39)	.001** Group 1 > group 3 Group 1 > group 4
AST (U/L)	142.40 ± 50.22 (96-267)	117.10 ± 14.93 (100-137)	111.10 ± 14.27 (90-132)	105.70 ± 15.44 (88-137)	.046* Group-1>Group-4
ALP (U/L)	233.30 ± 35.13 (157-279)	256.10 ± 60.35 (162-338)	281.30 ± 48.45 (170-320)	211.40 ± 33.84 (167-263)	.014* Group 4 > group 3
T3 (pg/mL)	1.5020 ± 0.46 (1.00-2.40)	2.0400 ± 0.39 (1.52-2.71)	1.1020 ± 0.19 (1.00-1.56)	1.5130 ± 0.38 (1.00-2.06)	.001** Group 2 > group 3
T4 (ng/dL)	1.0870 ± 0.21 (0.64-1.50)	1.3640 ± 0.15 (1.11-1.57)	1.0930 ± 0.10 (0,90-1,25)	1.1910 ± 0.17 (0.98-1.51)	.006** Group 2 > group 1 Group 2 > group 3
B12 (pg/mL)	664.00 ± 115.094 (494-847)	840.10 ± 85.728 (743-1048)	720.30 ± 73.071 (579-835)	723.10 ± 50.178 (636-796)	.001** Group 2 > group 1 Group 2 > group 3 Group 2 > group 4
Folic acid (ng/mL)	19.680 ± 8.509 (17.3-20.0)	20.000 ± 0.00000 (20.0-20.0)	19.960 ± 0.0966 (19.7-20.0)	19.990 ± 0.0316 (19.9-20.0)	.457
Ferritin (ng/mL)	6.360 ± 2.5242 (3.0-10.7)	6.300 ± 2.0067 (3.8-9.3)	8.400 ± 1.9994 (5.6-12.5)	7.330 ± 1.7398 (5.5-11.2)	.212
CRP (mg/L)	0.017 ± 0.01337 (0.01-0.05)	0.012 ± 0.0063 (0.01-0.03)	0.012 ± 0.0042 (0.01-0.02)	0.012 ± 0.0042 (0.01-0.02)	.710
IgE (ku/L)	0.1000 ± 0.00000 (0.10-0.10)	0.0660 ± 0.04427 (0.01-0.10)	0.1000 ± 0.00000 (0.10-0.10)	0.1000 ± 0.00000 (0.10-0.10)	.005** Group 2 < group 1 Group-2 < group 3 Group 2 < group 4

Kruskal–Wallis test and post hoc Dunn test with Bonferroni *P < .05, **P < .01.

ALP, alkaline phosphatase; ALT, alanine aminotransferase; AST, aspartate aminotransferase; CRP, C-reactive protein; HDL, high-density lipoprotein; Ig E, immunoglobulin E; LDL, low-density lipoprotein; T3, triiodothyronine; T4, thyroxine; TSH, thyroid-stimulating hormone; VLDL, very low-density lipoprotein.
